# Frontotemporal Dementia Caused by *CHMP2B* Mutations

**DOI:** 10.2174/156720511795563764

**Published:** 2011-05

**Authors:** A.M Isaacs, P Johannsen, I Holm, J.E Nielsen, FReJA Consortium

**Affiliations:** 1 Department of Neurodegenerative Disease, UCL Institute of Neurology, Queen Square, London, WC1N 3BG, UK; 2 Department of Cellular and Molecular Medicine, The Panum Institute, University of Copenhagen, Denmark; 3 Memory Disorders Research Unit, Department of Neurology, Copenhagen University Hospital, Rigshospitalet, Copenhagen, Denmark; 4 Department of Pathology, Randers Hospital, Randers; 5 Laboratory for Experimental Neuropathology, Danish Neuroscience Center, Aarhus University Hospital, Aarhus, Denmark; 6 The FreJA (Frontotemporal Dementia Research in Jutland Association) consortium: Elizabeth Fisher^1^, Martin Rossor^1^, Anders Gade^2^, Tove Thusgaard, ^2^Susanne Gydesen, Psychiatric Centre Ballerup, Copenhagen University Hospital, Ballerup, Denmark. Elisabet Englund, Department of Pathology, University Hospital, Lund, Sweden. Jerry Brown, Department of Neurology, Addenbrooke’s Hospital, Cambridge. John Collinge^1^, MRC Prion Unit UCL Institute of Neurology, London, UK

**Keywords:** Frontotemporal dementia, CHMP2B, endosome, lysosome, autophagy, brain imaging, neuropathology.

## Abstract

*CHMP2B* mutations are a rare cause of autosomal dominant frontotemporal dementia (FTD). The best studied example is frontotemporal dementia linked to chromosome 3 (FTD-3) which occurs in a large Danish family, with a further *CHMP2B* mutation identified in an unrelated Belgian familial FTD patient. These mutations lead to C-terminal truncations of the CHMP2B protein and we will review recent advances in our understanding of the molecular effects of these mutant truncated proteins on vesicular fusion events within the endosome-lysosome and autophagy degradation pathways. We will also review the clinical features of FTD caused by *CHMP2B* truncation mutations as well as new brain imaging and neuropathological findings. Finally, we collate the current data on *CHMP2B* missense mutations, which have been reported in FTD and motor neuron disease.

## INTRODUCTION

Frontotemporal lobar degeneration (FTLD) comprises a heterogeneous group of disorders which are all characterised by gross atrophy primarily of the frontal and/or temporal lobes. FTLD generally presents with either personality change, termed behavioural variant frontotemporal dementia (bvFTD) (or simply FTD) or distinct language impairments termed language variant FTD (lv-FTD), including semantic dementia (SD) and progressive non-fluent aphasia (PNFA) [1, 2]. FTLD can also be associated with parkinsonism, or with motor neuron disease, which is termed FTD-MND [3]. FTLD also has clinical and neuropathological overlap with the atypical parkinsonian movement disorders corticobasal degeneration (CBD) and progressive supranuclear palsy (PSP) [4-7].

The neuropathology of FTLD syndromes is also heterogeneous. The two major pathologies are FTLD-tau and FTLD-TDP, which are characterised by tau or TDP-43 positive inclusions respectively [4, 8]. FUS-positive inclusions have recently been described in a subset of FTLD cases [9, 10], and two rare neuropathological subtypes remain: those with ubiquitin-positive inclusions that are negative for tau, TDP-43 and FUS, termed FTLD-UPS [11] (which are primarily, *CHMP2B* mutation cases), and those with no discernable inclusions, termed FTLD-ni [11].

The major genetic causes of FTLD are mutations in *GRN* [12, 13], which encodes progranulin and *MAPT *[14] which encodes tau, leading to FTLD-TDP and FTLD-tau respectively. Mutations in *TARDBP* (which encodes TDP-43) [15, 16], *VCP [17]*, *FUS* [18] and *CHMP2B* [19, 20] are much rarer genetic causes of FTLD. This review will focus on recent advances in the brain imaging, neuropathology, cell biology and genetics of FTLD caused by *CHMP2B* mutations.

## C-TERMINAL TRUNCATION MUTATIONS IN FAMILIAL FTD

The first mutation in *CHMP2B* was identified in frontotemporal dementia linked to chromosome 3 (FTD-3) [20], an autosomal dominant FTD which occurs in one large kindred from Denmark [20, 21]. The mutation occurs in the splice acceptor site for the 6^th^ and final *CHMP2B* exon, leading to the formation of two novel transcripts termed *CHMP2B^Intron5^* and *CHMP2B^Delta10^*. Both transcripts are present in the brains of FTD-3 patients: *CHMP2B^Intron5^* at ~35% and *CHMP2B^Delta10^* at ~10% the level of the wildytpe *CHMP2B* transcript [22]. The resulting proteins both lose the final 36 amino acids, which are encoded by exon 6, replacing them with either a single valine residue (CHMP2B^Intron5^) or a 29 amino acid nonsense sequence (CHMP2B^Delta10^) Fig. (**1**). A second *CHMP2B* mutation was subsequently identified in an autosomal dominant Belgian FTLD pedigree [19]. This mutation changes a glutamine residue at position 165 of the CHMP2B protein sequence to a stop codon and is therefore termed CHMP2B^Q165X^. This leads to a protein which lacks the final 49 amino acids Fig. (**1**). Importantly, it therefore appears that the mutations have a common mechanism - the deletion of the C-terminus of the protein [23].

## CLINICAL FINDINGS

The most comprehensive clinical picture of FTLD caused by *CHMP2B* mutation comes from the description of 22 FTD-3 patients [24] (of the 33 FTD-3 cases identified to date). Early personality change is the most common feature which can include less concern for others, an unkempt appearance, disinhibition, inappropriate emotional responses and restlessness which later can be accompanied by aggression. Apathy can also occur as the disease progresses. Hyperorality is also common, encompassing mouthing of non-food objects, over-eating of sweet foods and chain smoking. Early dyscalculia was observed in 8 of the 22 patients and was the presenting symptom in one case. Sterotyped behavioural routines were observed in 11 of the 22 patients. For example, one patient continuously walked round the outside of her house and another repeatedly turned objects upside down to inspect their bases. Apraxia is also common with patients unable to copy gestures. A progressive aphasia develops during the course of the disease, although normally not with features of PNFA or SD. It is characterised by reduced spontaneous speech with relative preservation of reading and repetition, most consistent with a dynamic aphasia. An overt motor syndrome develops late in the disease course which can include parkinsonian features, dystonia, pyramidal signs and myoclonus, ultimately leaving the patients bedridden. None of the patients have had any clinical signs of either upper or lower motor neuron impairment, and no neurophysiological EMG studies have been done so far in the Danish FTD-3 patients. A new branch of the FTD-3 family was recently identified and clinical descriptions of a further two cases were similar to those previously reported [21]. The average age of onset for FTD-3 is 58 years of age and the mean duration is 10 years. Ongoing neuropsychological studies indicate that some of the patients can have impaired memory function in the earlier phases of the disease, but this is not a prominent feature and most often not a subjective complaint by the patients.

The clinical picture of the Belgian *CHMP2B* mutation patient has some similarities to the FTD-3 cases including loss of decorum, mild disinhibtion and severe dyscalculia early in the disease course [19]. The presenting symptom was dysgraphia [19] which has not been reported in any FTD-3 case.

## BRAIN IMAGING IN FTD-3

### Structural Imaging

In patients with clinical FTD-3 disease computerised tomography (CT) scans have been performed in 9 cases [21, 24] and MRI has been reported in one case [24], with scans performed on 3 further patients (our unpublished data). At diagnosis the scans shows a generalised central and cortical atrophy. There is no anterior or lateral preponderance. The posterior central atrophy might be more pronounced although the number of patients studied has been limited [24]. MRI on one patient has shown some non-specific white matter hyper intensities on T2 and Flair Imaging, which were considered most likely not directly related to FTD-3. A generalised atrophy was observed which was most marked in the frontal parietal and occipital lobes [24].

MRI in presymptomatic *CHMP2B* mutation carriers compared to first degree relatives without the mutation indicated that a global [25] as well as focal cortical atrophy [26] can occur before onset of symptoms.

Two CT scans have been performed on the Belgian *CHMP2B* mutation positive case [19]. The first scan was performed 2 years after symptom onset and showed mild frontal atrophy. A second CT scan 6 years into the disease course showed a generalised atrophy.

### Functional CBF Imaging in FTD-3

Measurements of regional cerebral blood flow (rCBF) with H_2_^15^O positron emission tomography (PET) has been performed in 3 FTD-3 patients with MMSE ranging from 0-24 points [21, 24]. The rCBF was normal in the primary visual cortex, thalami, basal ganglia, cerebellum, and in the more mild patients also in a small area of the right lateral frontal cortex. The rCBF of all other cortical regions was severely impaired with the most prominent flow deficits in the parietal, anterior-frontal, and lateral temporal cortices. Representative MRI and PET scans are shown in Fig. (**2**).

## NEUROPATHOLOGY

FTD-3 brains show a global cortical atrophy, which is most marked in the frontal cortex but also prominent in the temporal cortex; the parietal lobe can also be affected but the cerebellum is spared [24, 27]. There is no obvious atrophy of the hippocampus, amygdala, basal ganglia or substantia nigra [24, 27]. Cortical neuronal loss is accompanied by gliosis and microvacuolisation [24, 27]. Ubiquitin- and p62-positive neuronal cytoplasmic inclusions are observed in FTD-3 brains in the dentate granule cell layer of the hippocampus and to a lesser extent in the frontal cortex [27]. These inclusions are negative for both TDP-43 [27] and FUS [28], classifying FTD-3 as FTLD-UPS. We recently reported that two familial FTLD-UPS cases were negative for CHMP2B mutation [29], suggesting that although FTLD-UPS is a rare FTLD pathology it has more than one genetic cause.

The neuropathology of FTD-3 has been further refined by the discovery of enlarged vacuoles in cortical neurons [22]. The vacuoles were present in the frontal, temporal, parietal and occipital cortices, but not in the cerebellar cortex, a pattern generally reflecting the atrophy observed by neuropathology and brain imaging. The vacuoles were positive for a marker to the late endosome, suggesting they could be aberrant enlarged late endosomes. Enlarged endosomes have not been described in other FTLD cases to date, although enlarged early and late endosomes have been observed in cortical neurons in Down’s syndrome and Alzheimer’s disease brains [30-32].

FTD-3 brains have no abnormal aβ, α-synuclein, prion protein or neurofilament staining [27]. Some tau pathology has been observed, but at insufficient levels for a diagnosis of FTLD-tau. Tau positive inclusions were identified in the frontal cortex of three FTD-3 brains, but at levels that could be explained by normal aging [33]. Tau staining in further brain regions of the same cases, as well as one additional case, revealed neurofibrillary tangles, in the absence of amyloid deposits, in the hippocampus, entorhinal cortex and transentorhinal cortex consistent with Braak stage IV [27]. This distribution of neurofibrilliary tangles in the absence of amyloid pathology is typical of tangle predominant dementia [34, 35], but such cases are clinically distinct from FTD-3, presenting with an Alzheimer’s disease-like dementia with very late onset (80-90 years of age) [34, 35].

## FUNCTIONAL STUDIES

CHMP2B is part of the multi-protein ESCRT-III complex (endosomal sorting complex required for transport-III). ESCRT-III has a role in two protein degradation pathways that converge on the lysosome: the endosome-lysosome pathway and autophagy. The CHMP2B C-terminal truncation mutants can impair both of these pathways and each will be discussed.

### Endosome-Lysosome Pathway

The endosome-lysosome pathway is responsible for the degradation of endocytosed proteins such as transmembrane proteins and cell surface receptors. The proteins are endocytosed into early endosomes which mature into late endosomes and then fuse with lysosomes, allowing protein degradation to occur. Over-expression of CHMP2B^Intron5^, CHMP2B^Delta10^ or CHMP2B^Q165X^ in human neuroblastoma cells leads to the formation of enlarged late endosomes [19], suggesting an impairment of this pathway. The relevance of this cell culture data was confirmed by the observation of aberrant enlarged late endosomes in fibroblasts from both FTD-3 patients and the Belgian *CHMP2B* mutation patient, as well as the identification of enlarged endosomes in cortical neurons in FTD-3 patient brain [22]. Further cell culture data indicates that this defect is due to the C-terminal CHMP2B mutants impairing the fusion of endosomes with lysosomes [22]. The late endosomes in CHMP2B mutant cells were impaired in their ability to recruit Rab7 [22], a key member of the endosome-lysosome fusion machinery [36]. *RAB7* mutations have been identified in the rare motor and sensory neuropathy Charcot-Marie-Tooth type 2B [37, 38], suggesting altering Rab7 function can lead to neurodegenerative disease.

How impairment of endosome-lysosome fusion leads to disease is still not clear. The ESCRT complex mediates the lysosomal degradation of growth factor receptors [39] and neurotransmitter receptors [40, 41], both of which are essential for neuronal maintenance and function. CHMP2B^Intron5^ expression can also activate the Toll-like receptor pathway [42], which has been implicated in neurodegeneration [43]. Further research, including the development of mouse models, will be required to determine how these different pathways contribute to the neuronal cell death caused by *CHMP2B* mutation.

### Autophagy

Autophagy is a cellular system that can degrade damaged organelles, long-lived proteins and protein aggregates [44]. The proteins or organelles to be degraded are encapsulated by autophagosomes, which either fuse directly with lysosomes, or first with endosomes forming a hybrid organelle termed an amphisome, which then fuses with the lysosome [45, 46]. Expression of CHMP2B C-terminal truncation mutants leads to an accumlation of autophagosomes, due to their impaired fusion with endosomes and/or lysosomes [47, 48]. Rab7 is also required for autophagosome-lysosome fusion [49], suggesting a similar mechanism could be responsible for impairing vesicular fusion events in both the autophagy and endosome-lysosome pathways. An accumulation of autophagosomes in affected neurons has been observed in Alzheimer’s disease [50, 51], Parkinson’s disease [52], and prion diseases [53-57], and deletion of key autophagy genes leads to neurodegeneration in mice [58, 59]. Deciphering the precise mechanism by which mutant CHMP2B affects autophagy may therefore have broad relevance for understanding neurodegenerative disease mechanisms.

## MISSENSE MUTATIONS IN FTD-MND SPECTRUM DISORDERS

Five missense mutations Fig. (**1**) in eight individuals have been identified in *CHMP2B* in a range of FTD-MND spectrum disorders (Table **1**). A recent study screened 433 MND patients for *CHMP2B* mutations and identified 3 distinct *CHMP2B* missense mutations in four patients. Interestingly, all 4 patients suffered from primary muscular atrophy (PMA), a form of motor neuron disease which predominantly affects the lower motor neurons [60]. 40 of the 433 MND cohort were PMA variant cases suggesting that *CHMP2B* mutations could account for 10% of such cases. A previous study found no *CHMP2B* mutations in 538 MND cases [61], although it is unclear how many PMA cases were present in this cohort. Two of the four PMA cases had the same mutation, I29V, which has previously been identified in one case of FTD-MND [62] one case of FTD [63], but also in one control [64]. The final two PMA mutations, T104N and Q206H [60], have not been described in any other FTD or MND case. A further two missense mutations have been described, N143S, in a case of corticobasal degeneration [19], and D148Y in a case of semantic dementia [20].

Three of the eight missense mutation cases have a family history (Table **1**) but unfortunately samples of family members were not available to confirm segregation of the mutations. In order to confirm pathogenicity of the missense mutations it will be important to show that they segregate with disease in familial cases. Further clarification is also needed on the penetrance of the I29V mutation and the pathogenic mechanism of the missense mutations.

It is also intriguing that the PMA missense cases have TDP-43 pathology [60], whereas FTD-3 cases do not [27]. This could be explained by the missense mutations having a different effect to the gain of function C-terminal truncation mutations. Indirect evidence to support this possibility comes from an experiment where loss of function of the ESCRT-III complex due to knockdown of CHMP3 (also termed VPS24) led to the formation of cytoplasmic TDP-43 aggregates in cell culture, whereas expression of CHMP2B^Intron5^ did not [47]. The generation of knockout and missense mouse models may shed further light on this issue. In summary, further work is required to fully assess the role and importance of *CHMP2B* missense mutations in FTD and MND.

## CONCLUSIONS

The early clinical picture of FTD caused by *CHMP2B* mutations is similar to other behavioural variant FTD cases while the brain imaging and neuropathological findings are somewhat distinct. Despite being a rare cause of dementia, recent advances in our understanding of the molecular basis of *CHMP2B* mutations indicate that the mechanisms involved may be broadly relevant to neurodegenerative processes.

## Figures and Tables

**Fig. (1):**
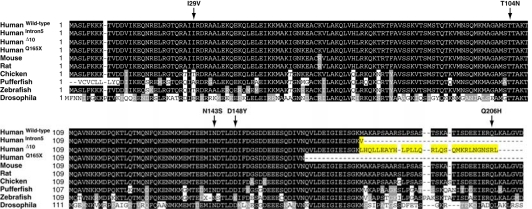
Multiple alignment of CHMP2B proteins. Wildtype CHMP2B and C-terminal truncations CHMP2B^Intron5^, CHMP2B^Delta10^ and CHMP2B^Q165X^ were aligned with CHMP2B homologues from other species. The novel C-termini of CHMP2B^Intron5^ and CHMP2B^Delta10^ are highlighted in yellow. CHMP2B missense mutations identified in FTD-MND spectrum disorders are arrowed.

**Fig. (2):**
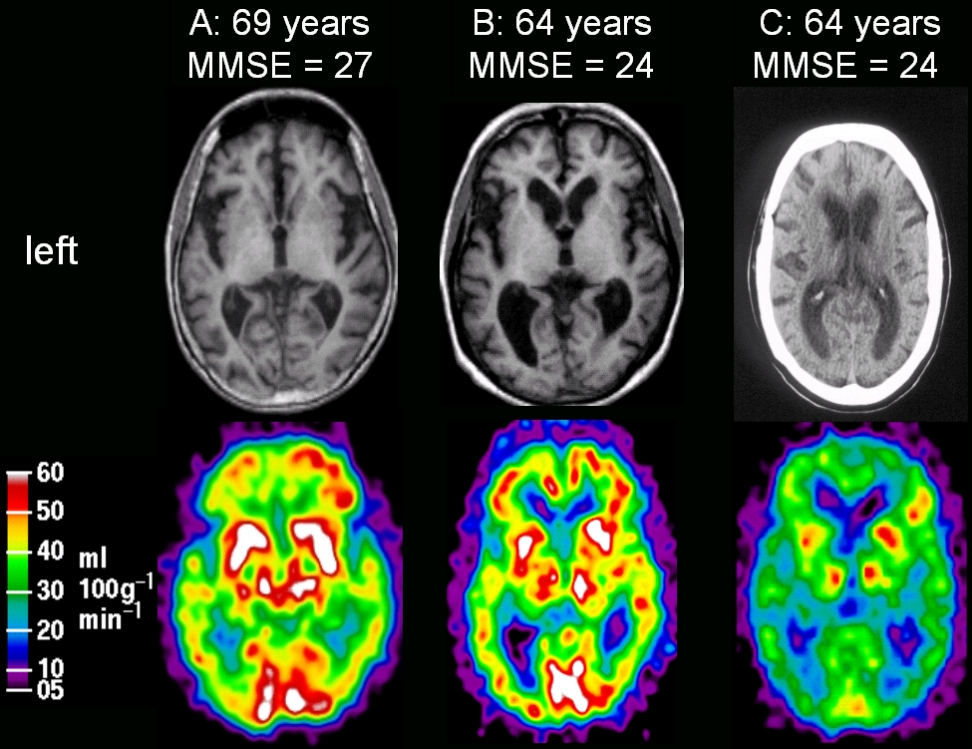
Representative brain scans in FTD-3 patients. Upper row: structural scans in FTD-3 with MRI for patients A and B, and a CT-scan for patient C. Lower row shows cerebral blood flow as measured by with H_2_^15^O -PET scanning for the same 3 patients.

**Table 1:** Cases Identified with *CHMP2B* Missense Mutations.

DNA Change	Protein Change	Disease	Family History	Controls Analysed in Study	Reference
c.85A>G	I29V	FTD	Y	190	[63]
c.85A>G	I29V	FTD-MND	Possible	640	[62]
c.85A>G	I29V	PMA	N	500	[60]
c.85A>G	I29V	PMA	N	500	[60]
c.311C>A	T104N	PMA	Y	500	[60]
c.428A>G	N143S	CBD	Y	459	[19]
c.442G>T	D148Y	SD	N	100	[20]
c.618A>C	Q206H	PMA	N	500	[60]

FTD-MND – Frontotemporal Dementia with Motor Neuron Disease. PMA – Primary Muscular Atrophy. CBD – Corticobasal Degeneration. SD – Semantic Dementia.

## ABBREVIATIONS

CBD: = Corticobasal degeneration
CHMP2B: = Charged multivesicular body protein 2B
CT: = Computerised tomography
ESCRT-III: = Endosmal sorting complex required for transport-III
FTD-3: = Frontotemporal dementia linked to chromosome three
FTLD: = Frontotemporal lobar degeneration
GRN: = Progranulin
Vps24: = Vacuolar protein for sorting 24
MAPT: = Microtubule associated protein tau
MND: = Motor neuron disease
MRI: = Magnetic resonance imaging
PET: = Positron emission tomography
PMA: = Primary muscular atrophy
PNFA: = Progressive non-fluent aphasia
SD: = Semantic dementia
TDP-43: = TAR DNA binding protein-43
